# A Rare Case of Isolated Cystic Hydatid of Thyroid Gland

**DOI:** 10.1097/MD.0000000000002929

**Published:** 2016-03-11

**Authors:** Huseyin Eken, Arda Isik, Gulhan Balci, Deniz Firat, Orhan Cimen, Mehmet Soyturk

**Affiliations:** From the General Surgery Department (HE, AI, DF, OC); Pathology Department (GB); and Radiology Department (MS), School of Medicine, Erzincan University, Erzincan, Turkey.

## Abstract

Hydatid cysts are most frequently localized within the liver and lungs, although they can also be found in highly vascularized tissues such as the brain, muscle, heart, pancreas, adrenal, and thyroid glands.

A 65-year-old female patient was admitted to our clinic with complaints of a progressively growing mass that was compressing the surrounding tissues and causing respiratory distress. The pathological result was obtained as cytic hydatid.

In patients with diagnosed hydatid cysts in the liver, systemic evaluation is necessary to rule out involvement of other organs. Among patients presenting with growths located in the neck, primary hydatid cyst of the thyroid gland must be considered in endemic regions.

## INTRODUCTION

The hydatid cyst is a parasitic disease caused by *Echinococcus granulosus*.^1^ Endemic areas include the Mediterranean countries, the Middle East, the southern part of South America, India, Iceland, Australia, New Zealand, and southern part of Africa. Echinococcosis is a rare disease in the United States and in northern Europe.^2^ Hydatid cyst is a common health issue in the Eastern and South-Eastern Anatolia Regions of Turkey, where livestock is common. Hydatid cyst is most frequently localized to the liver and lungs, but may be found in other highly vascularized organs such as the brain, muscle, heart, pancreas, adrenal, and thyroid glands.^3^ In patients with diagnosed hydatid cysts in the liver, systemic evaluation is necessary to rule out involvement of other organs. Among patients presenting with growths located in the neck, primary hydatid cyst of the thyroid gland must be considered in endemic regions. Hydatid cysts in the thyroid may grow rapidly and result in compressive and obstructive problems. Also, rupture of hydatid cysts can trigger complications such as anaphylaxis, shock, and death.

## CASE

**FIGURE 1:**
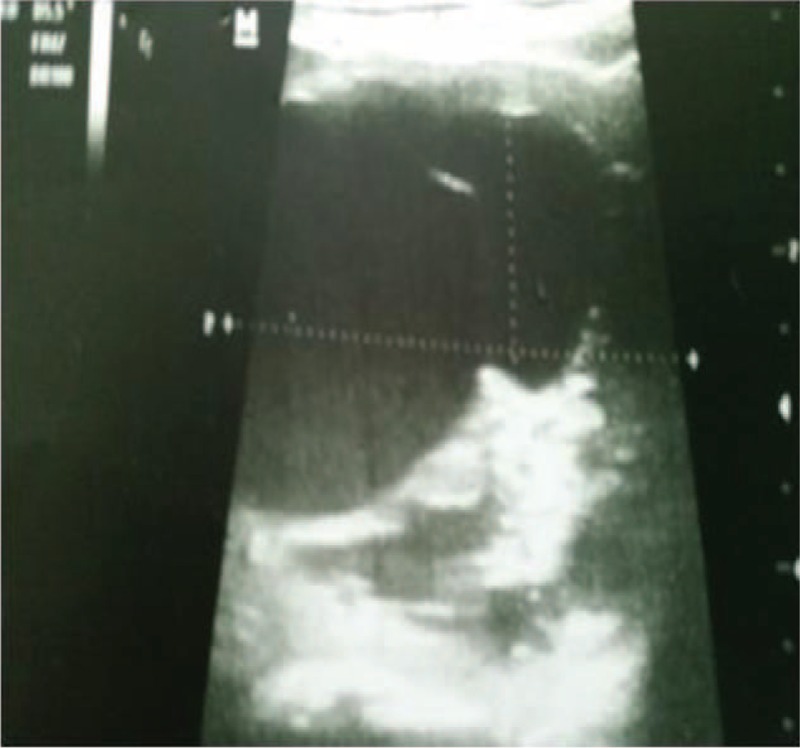
Ultrasonography of thyroid.

**FIGURE 2:**
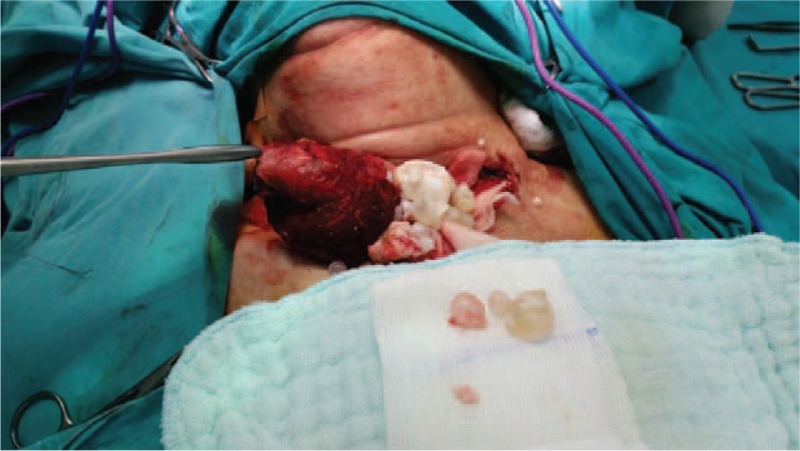
Intraoperative view.

**FIGURE 3:**
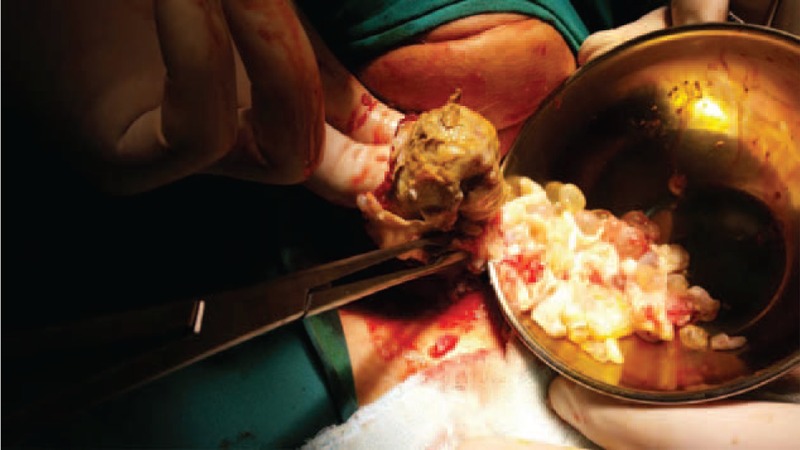
Intraoperative view.

A 65-year-old female patient was admitted to our clinic with complaints of a progressively growing mass that was compressing the surrounding tissues and causing respiratory distress. The patient's past medical history indicated progression of symptoms over the previous year. Physical examination revealed a 7 × 5 cm mobile, soft nodule in the left thyroid lobe. There was no lymphadenopathy in the neck. Physical examination was otherwise unremarkable. An ultrasonography scan showed that the right thyroid lobe measured 21 × 17 mm and had several hypo-echoic nodules, the biggest measuring 8 × 6 mm. The left thyroid lobe measured 71 × 52 mm and had internal echogenities with thick walls and thick septations in which concentrated contents sank to the bottom of the cystic lesion in a multicystic and multiloculated manner (complex cystic nodule) (Figure 1). Before obtaining the biopsy results, the patient underwent surgery to treat respiratory distress. During the operation, the cystic structures opened unintentionally and the germinative membrane and daughter vesicles were observed (Figures 2 and 3). Identifying the structure as a hydatid cyst, the operational team decided to perform a total bilateral thyroidectomy. The postoperative histopathological examination was consistent with hydatid cyst. The presence of other hydatid cyst foci was evaluated after the surgery using abdominal and thorax tomography scans, and serodiagnostic techniques. The indirect hemagglutination test and the enzyme-linked immunosorbent assay (ELISA) were both positive. In the laboratory, no eosinophilia and hypogammaglobulinemia were detected. The radiologic evaluations showed no further signs of hydatid cyst. The patient had no postoperative complications and was discharged. She was prescribed albendazol treatment as 3 weeks oral doses of 10 mg/kg/day separated by 1-week intervals.

No signs of recurrence by serology and ultrasonography were seen during the 3 years of follow-up time.

## DISCUSSION

Hydatid cyst occurs frequently in Turkey and may affect as many as 1/2000 patients.^2^ The majority of the patients reside in rural regions and work closely with livestock. The causative agents of the disease are parasites, most frequently *E. granulosis* and *E. alveolaris*. The parasite typically resides in the small intestines of definitive hosts. These are carnivores such as dogs, foxes, wolves, coyotes, lynxes, cats, and black bears. Intermediate hosts are sheep, rodents, and cows. The infective eggs are released to the environment through feces. Transmission to humans occurs through the ingestion of echinococcus eggs by water, foods, and direct contact with dogs. Embryos pass into the digestive system and migrate along the wall of the intestine, eventually passing into the portal circulation. These embryos primarily settle in the liver (60%). The remaining embryos pass into the microcirculation and settle in the lungs (30%), brain, muscle, heart, pancreas, adrenal, and thyroid glands.^4^ Cysts may occur in several organ systems simultaneously. In patients with diagnosed hydatid cyst of the liver, systemic investigations must rule out involvement of other organs. The rate of multiorgan involvement is reported to be 20% to 30%.

Atypical localization of hydatid cysts in the thyroid gland is rare. The frequency of these cysts is reported to be 0.5% to 1% in Turkey. The total number of cases reported in the literature is 160.^5,6^ Although blood flow to the thyroid gland is strong, the narrow thyroid arteries and angle perpendicular to the carotid generally prevent settling of hydatid cysts.^4^ Hydatid cyst of thyroid usually occurs as a solitary and primary infection, although some cases with accompanying hydatid cysts in the liver or lung have been reported in the literature.^7^

The diagnosis of echinococcosis is made based on history, physical examination, radiologic imaging methods, serological tests, and aspirations. The indirect hemagglutination test and ELISA have a sensitivity of 80% overall (90% in hepatic echinococcosis, 40% in pulmonary echinococcosis) and are the initial screening tests of choice. The ELISA test is useful in follow-up to detect recurrence. A hydatid cyst of the thyroid gland is a progressively growing, solitary nodule limited to a single lobe observed as a cold nodule in scintigraphy. It may be confused with thyroid cancers, which also appear as cold nodules in scintigraphy. Diagnosis is made upon visualization of the germinal vesicles in ultrasonography or with the assistance of tomography and magnetic resonance imaging. Fine needle aspiration biopsy is not advised since it may cause the spread of cystic fluid and possibly anaphylactic shock.^8,9^ Hydatid cyst of the thyroid is usually asymptomatic. The diagnosis is usually made incidentally during imaging for other reasons. Hydatid cysts may grow and compress surrounding tissues and become symptomatic, causing respiratory distress, dysphagia, compression of recurrent laryngeal nerve, involvement of strep muscle, perforation of the cyst into the trachea, and death as a result of allergic reactions.^10^

Hydatid cysts of the thyroid are typically first identified during surgical operation. Our patient was admitted with long-term complaints of a lump in the neck, dysphagia, and finally respiratory distress. Gürses et al^11^ reported a hydatid cyst of thyroid gland with a similar appearance in ultrasonography and associated with a type I hydatid cyst of the liver. In contrast to that, Dettori et al^4^ reported that preoperative ultrasonography does not assist with diagnosis. The radiologic signs were not specific at initial admission in the present case. Diagnosis of our patient was made during the operation. The definitive diagnosis of hydatid cyst of thyroid gland is made upon histopathological examination. Under a light microscope, in slides stained with hematoxylin and eosin, we observed a fibrous capsule containing an acellular, laminated cuticular layer with a central germinal membrane and scolex.

As with all hydatid cysts, the primary treatment of hydatid cysts of the thyroid gland is surgery. The risk of disseminating cystic fluid and subsequent anaphylaxis must be considered and surgeons should proceed with great care. Chemotherapy in the postoperative period is essential for preventing recurrence.^8^ For chemotherapy, there are other therapeutic options, for example, mebendazole and praziquantel. Albendazole seems to be ineffective in the treatment of primary liver cysts in surgical candidates.

## CONCLUSIONS

The present case illustrates the presence of hydatid cysts in atypical anatomical locations in endemic regions. Hydatid cysts must be considered when evaluating cystic nodules of the thyroid gland. We hope that this rare example may provide a useful illustration of atypical signs and symptoms of hydatid cysts.

## References

[1] Sorogy ME El-Hemaly M Aboelenen A Pancreatic body hydatid cyst: a case report. *Int J Surg Case Rep* 2014; 6:68–70. 25528027 10.1016/j.ijscr.2014.11.062 PMC4337928

[2] Sayek I Onat D Diagnosis and treatment of uncomplicated hydatid cyst of the liver. *World J Surg* 2001; 25:21–27. 11213152 10.1007/s002680020004

[3] Bedioui H Chebbi F Ayadi S Primary hydatid cyst of the pancreas: diagnosis and surgical procedures. Report of three cases. *Gastroenterol Clin Biol* 2008; 32:102–106. 18405655 10.1016/j.gcb.2007.12.014

[4] Dettori G Madeddu G Marongiu G Echinococcosis of the thyroid gland: two new cases. *Am Surg* 1980; 46:530–533. 7416635

[5] Chetty R Crovve P Cant P An unusual thyroid cyst. *S Afr J Surg* 1991; 29:158–159. 1763396

[6] Ozerkan E Gurcınar M Sarıoglu B A case of cystic echinococcosis in thyroid gland: a very rare localisation of echinococcosis infection. *Turk J Endocrinol Metabol* 1999; 4:181–183.

[7] Al-Qassab KH Abdul-Rahman H Safar S Hydatid disease of the thyroid. *Int Surg* 1982; 67:435–436. 7183603

[8] Köksal AŞ Arhan M Oğuz D Kist Hidatik. *Güncel Gastroenterol* 2004; 8:61–67.

[9] Erbil Y Barbaros U Başpinar Hydatid cyst of the thyroid gland: two case reports. *Infect Dis Clin Pract* 2005; 13:318–320.

[10] Chandara T Prakash A A case of hydatid cyst of thyroid. *Br J Surg* 1965; 52:235–237. 14261138 10.1002/bjs.1800520324

[11] Gürses N Baysal K Gürses N Hydatic cyst in the thyroid and submandibular salivary glands in a child. *Z Kinderchir* 1986; 41:362–363. 3825303 10.1055/s-2008-1043379

